# Patients with severe odontogenic infections receive insufficient dental treatment before hospitalization – a retrospective cross-sectional study

**DOI:** 10.2340/aos.v83.42371

**Published:** 2024-12-20

**Authors:** Rasmus Søndenbroe, Merete Markvart, Isabel Diaz-Pines Cort, Blaine Gabriel Fritz, Claus Henrik Nielsen, Thomas Bjarnsholt, Sanne Werner Møller Andersen, Simon Storgård Jensen

**Affiliations:** a Section of Oral Biology and Immunopathology, Department of Odontology, University of Copenhagen, Copenhagen, Denmark; b Section of Clinical Oral Microbiology, Department of Odontology, University of Copenhagen, Copenhagen, Denmark; c Costerton Biofilm Centre, University of Copenhagen, Copenhagen, Denmark; d Institute for Inflammation Research, Copenhagen University Hospital, Copenhagen, Denmark; e Department of Clinical Microbiology, Copenhagen University Hospital, Copenhagen, Denmark; f Department of Oral and Maxillofacial Surgery, Copenhagen University Hospital, Copenhagen, Denmark

**Keywords:** Severe odontogenic infections, hospitalization, preventive dentistry

## Abstract

**Objectives:**

The aim was to provide an in-depth characterization of patients hospitalized with severe odontogenic infections (SOI), especially in relation to the origin of the infection. Furthermore, the aim was to generate an overview of which kind of treatment the patients had received before hospitalization and to analyze risk factors for prolonged length of hospital stay.

**Material and methods:**

The study was a retrospective cross-sectional study, which included patients hospitalized at the University Hospital of Copenhagen, Denmark, with SOI from November 2012 through 2019. Data were extracted from medical hospital records. Analysis was performed using the χ^2^ test, analysis of variance, multiple correspondence analysis (MCA), and logistic regression.

**Results:**

A total of 384 eligible patients were included. The most frequent origin of infection was apical periodontitis (46.9%), infection after tooth extraction (25.8%), multiple infectious foci (8.6%), and pericoronitis (6.0%). Significant differences in concomitant diseases (*p* = 0.017) were found between the groups of origin of infection. The MCA model showed little to no ability to generate an in-depth characterization of the group of patients. Eleven patients (2.9%) were treated with incision and drainage before hospitalization, and 131 patients (34.3%) received no kind of antibiotic before hospitalization.

**Conclusion:**

The results indicate that clusters of variables could not be related to the origin of infection. In general, patients received insufficient treatment before hospitalization. Future studies should define risk factors for developing SOI and examine dental records of dental treatment before hospitalization.

**Clinical relevance:**

To improve prehospital treatment with patients with SOI, general dental practitioners should treat the origin of the infection, attempt drainage, and optimize the prescription of antibiotics.

## Introduction

An odontogenic infection can develop, worsen, and spread into the maxillofacial and parapharyngeal spaces, becoming a severe odontogenic infection (SOI). If left untreated, SOI can lead to severe morbidity and even mortality, and treatment in a hospital setting is indicated [1–5]. Hospitalization with SOI can include treatment with intravenous antibiotics, surgery in general anesthesia, and long-term hospitalization. SOI may thus have severe consequences for individual patients. Additionally, these patients utilize a substantial number of resources in the health care system.

Odontogenic infections are relatively frequent and can in most cases be successfully resolved through treatment in general dental practice [6, 7]. The treatment of an oral infection may be relatively simple and effective by eliminating the causative factor. If an abscess has developed, incision and drainage may be performed, and antibiotic treatment can be prescribed if the patient’s general condition is affected. However, most patients hospitalized with SOI have not received appropriate treatment in general dental care [8].

Studies have shown that patients suffering from SOI constitute a heterogeneous group, varying in age, general health (number of comorbidities), and origin of infection [8–15]. To define preventive measures aimed at avoiding SOI requiring hospitalization, an in-depth understanding of the general characteristics and anamnesis of patients is essential. Furthermore, a better understanding of the origin of the infection in patients with SOI could add significantly to the existing literature [16] and contribute to the prevention of SOI. Thus, the overall aim of this study is to identify factors that lead to SOI and subsequent hospitalization. A better understanding of these factors may allow the initiation of preventive measures.

### Hypothesis

The primary hypothesis was that patients with different origins of infection vary significantly in terms of sex, age, medical history, and course of hospital stay. A secondary hypothesis was that patients with SOI generally receive insufficient dental and medical treatment for their odontogenic infection before hospitalization.

## Method

### Setting

This is a cross-sectional study of data on patients hospitalized with SOI between the 1st of November 2012 and 31st December 2019. All patients hospitalized with SOI at the Department of Oral and Maxillofacial Surgery, Copenhagen University Hospital, Denmark, were eligible for inclusion into the study.

### Inclusion of patients

Patients were included if the following four criteria were met: (I) diagnosed with abscess or phlegmone in the mouth (WHO ICD-10 diagnosis DK12.2 and K12.2), (II) the origin of infection was odontogenic, (III) the patient was hospitalized at least one night, and (IV) sufficient medical records were present.

### Data source

Data from eligible patients were collected through the electronic medical record system (Electronic Health Records, EPIC). The search was performed among all patients hospitalized with a diagnosis of abscess or phlegmone in the mouth (WHO ICD-10 code DK12.2 and K12.2) and at least one overnight stay at the hospital.

The first author (Rasmus Søndenbroe) reviewed medical records in consultation with two maxillofacial surgeons (Simon Storgård Jensen, Sanne Werner Møller Andersen). Data entry was performed using EXCEL 2016 (Microsoft).

### Variables

The primary variable of interest was the origin of infection. The origin of infection was determined by analysis of clinical presentation, radiographic findings, and any dental treatment performed immediately before hospitalization.

Anamnestic information included the duration of symptoms, dental treatment, antibiotic treatment, and the professional background of the referring health care professional.

Information on sex, age, comorbidities, medication intake, smoking, and alcohol consumption was collected from medical records. Smoking and alcohol intake were recorded as binary variables with the categories yes/no. Systemic diseases of interest were diabetes mellitus (DM), cardiovascular diseases (hypertension, hypercholesterolemia, other cardiovascular diseases (OCVD)), psychiatric disorders, immunodeficiency diseases, chronic obstructive pulmonary disease (COPD), asthma, and osteoporosis. The disease was recorded by their presence.

Data on the course of treatment during hospitalization included records of operative treatment categorized into local and general anesthesia, administration of antibiotics, paraclinical test results (inflammatory markers, microbiologic sampling, antibiotic susceptibility, radiographic evaluation), and length of hospital stay (LOS). In patients attending control visits, dysesthesia was recorded. Patients who developed cervical necrotizing fasciitis (CNF) were registered following the procedure described in the article by Hansen et al. [3].

### Radiographic assessment

Dental status and dental pathological conditions were recorded by panoramic radiographs (PR). Decayed, missing, and filled teeth (DMFT) along with apical and peri-coronal radiolucency were determined by the principal investigator (RS). Dental decay was defined as radiolucency within the depth of at least one third of dentine and categorized into, respectively, 0, 1–3, 4–10, and 11–28 lesions. Number of teeth with apical periodontitis (AP) were categorized into 0, 1, and 2–10. Periodontal bone loss was evaluated in accordance with the guidelines of the PAROKRANK study [17] and categorized into ≥80%, 66% to 79%, and <66% mean remaining bone.

The radiographic evaluation was calibrated with a dentist (Merete Markvart) and maxillofacial surgeons (SSJ/SWMA), with whom diagnostic doubts were also discussed. Spread of infection to adjacent anatomical spaces was determined based on radiological reports of computerized tomography (CT) scans.

### Statistical analysis

The final dataset included 255 different variables. Descriptive and analytic statistics were performed in Statistical Package for the Social Science (SPSS) version 28.0.0.0. SPSS. Statistical differences in the distribution of explanatory variables between groups were analyzed using the χ^2^ test on categorical variables. Where statistically significant differences were detected, adjusted standardized residuals were used to compare the expected and the observed distribution between the groups of origin of infection. Analysis of variance (ANOVA) was used to analyze differences in continuous variables. Shapiro-Wilk tests were applied to test the normal distribution of these variables, and the Kruskal-Wallis tests were used for nonparametric testing. Bonferroni corrections were performed in the ANOVA tests.

A logistic regression model was used to determine risk factors for prolonged LOS at the hospital. The LOS was divided into two groups, respectively, <4 days and prolonged LOS at ≥4 days. Potential risk factors for a prolonged LOS were chosen by the results of other studies, and all comorbidities were perceived as potential risk factors. These potential risk factors were tested in a univariate test, and the potential risk factors that affected LOS were included in the multivariate regression model. The overall adequacy of the logistic regression model was tested using Omnibus Tests of Model Coefficients and Hosmer and Lemeshow goodness of fit test. The ability of the models to explain the variability in data was tested according to Nagelkerke *R^2^*. The significance level was set at *p* < 0.05 for all tests.

A separate analysis was performed to identify clusters of variables. Given the high dimensionality of the data, a dimensionality reduction technique was applied to uncover general patterns. As most variables were categorical, multiple correspondence analysis (MCA) was chosen as an approach for dimensionality reduction. Before MCA, variables with more than 10% missing values and patients with remaining missing values for any variable were excluded from the dataset. MCA was performed in *R* version 4.2.2 using the libraries FactoMineR and factoextra.

## Results

**Figure 1:**
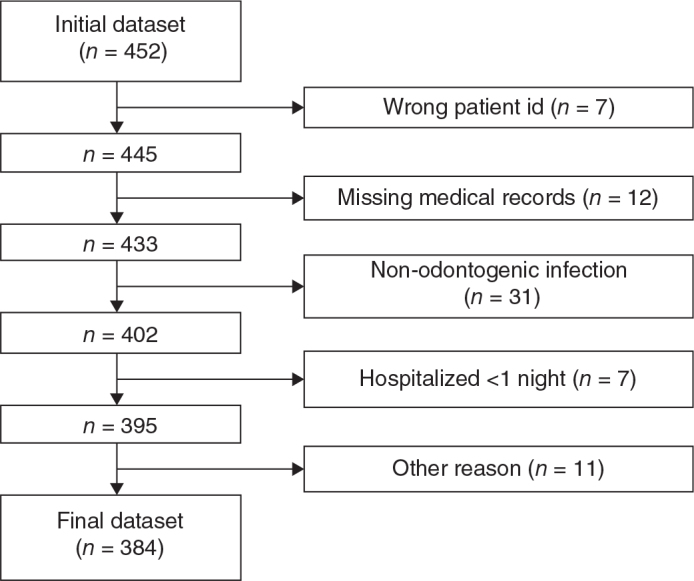
Flow chart of exclusion. This figure illustrates the exclusion of patients following the exclusion and inclusion criteria.

An initial search in the medical records database resulted in 452 patients. Out of these, 68 patients did not fulfill the inclusion criteria and had to be excluded, resulting in a study population of 384 unique patients (Figure 1).

### Clinical characteristics

In patients with a single origin of infection, the most common causes were AP (46.9%), followed by post-extraction infection (PEI) (25.8%) and pericoronitis (PERI) (6%) and patients with multiple foci (MF) (8.6%) (Table 1). Patients with unknown foci (3.7%), osteonecrosis (2.6%), infection related to osteosynthesis material (2.3%), periodontitis (1.5%), cysts (1.3%), fractures (0.5%), and osteomyelitis (0.5%) were categorized into a 5th group named Other (Table 1).

**Table 1:** General health information.

Characteristic	Origin of the infection												
	Total		AP		PEI		PERI		MF		Other		p-value
	*n*	%	*n*	%	*n*	%	*n*	%	*n*	%	*n*	%	
Patients	384	100.0	180	100.0	99	100.0	23	100.0	33	100.0	49	100.0	
Age, years, mean ± s.d.	43.9	19.5	42.7	19.6	41.2	19.1	40.3	16.7	51.4	17.1	50.2	20.6	0.009*
Gender, male	182	47.4	80	44.4	43	43.4	14	60.9	20	60.6	25	51.0	0.240
Smoking	130	33.9	69	38.3	26	26.3	7	30.4	17	51.5	11	22.4	0.010*
Alcohol consumption	183	47.7	83	46.1	47	47.5	7	30.4	21	63.6	25	51.0	0.153
Comorbidites	149	38.8	74	41.1	27	27.3	7	30.4	16	48.5	25	51.0	0.034*
Psyciatric disorder	61	15.9	32	17.8	13	13.1	3	13.0	5	15.2	8	16.3	0.877
Hypertension	46	12.0	25	13.9	8	8.1	3	13.0	5	15.2	5	10.2	0.663
Hypercholesterolemia	14	3.6	8	4.4	4	4.0	0	0.0	2	6.1	0	0.0	0.474
OCVD	26	6.8	11	6.1	7	7.1	1	4.3	4	12.1	3	6.1	0.781
DM	27	7.0	15	8.3	4	4.0	1	4.3	4	12.1	3	6.1	0.522
Autoimmune disease	9	2.3	2	1.1	4	4.0	0	0.0	2	6.1	1	2.0	0.183
COPD	23	6.0	10	5.6	4	4.0	0	0.0	0	0.0	9	18.4	0.001*
Asthma	22	5.7	12	6.7	5	5.1	0	0.0	0	0.0	5	10.2	0.232
Medication, mean ± s.d.	2.0	3.4	3.5	3.4	1.5	3.3	1.1	1.3	2.9	4.2	3.2	3.9	0.021*
Antiresorp. or radio.	20	5.2	6	3.3	3	3.0	3	13.0	0	0.0	8	16.3	0.001*
Penicillin allergy	33	8.6	18	10.0	10	10.1	0	0.0	3	9.1	2	4.1	0.373

Test results of chi-square, Shapiro-Wilk, or Kruskal-Wallis with significant levels < 0.05 indicated by *.

AP: apical periodontitis; PEI: post extraction infection; PERI: Pericoronitis, Antiresorp, or radio (antiresorptive therapy or radiotherapy); MF: multiple foci; COPD: chronic obstructive pulmonary disease; DM: diabetes Mellitus; OCVD: other cardiovascular diseases; s.d.: standard deviation.

Patients with MF had a higher mean age (51.4 years), compared to the entire study population (43.9 years (*p* = 0.009)). There was a significantly higher proportion of smokers in the group of patients with MF compared to the other groups (*p* = 0.01). Significantly more patients in the three groups AP, MF, and Other suffered from comorbidities (*p* = 0.034). The number of patients who had previously received antiresorptive therapy or radiotherapy was relatively low (5.2%), but a significant difference was found between the group Other and the rest of the groups (*p* = 0.001). Furthermore, the number of medications was significantly higher in the groups AP, MF, and Other compared to PEI and PERI (*p* = 0.021) (Table 1).

The number of patients who had incision and drainage performed before hospitalization was relatively low (*n* = 11), and no significant difference was found between the five diagnosis groups (Table 2). Patients with PERI were more likely to have been initially misdiagnosed than the other four groups (*p* = 0.002) and were most often mistaken for peritonsillar abscesses. The mean duration of symptoms was found to be 6.5 days. Of all patients, 35.4% were prescribed penicillin, and 27.6% were prescribed metronidazole before hospitalization, with no difference between the diagnosis groups (Table 2). Of all patients, 13.3% received metronidazole in combination with penicillin, while 34.6% had no prescription for any antibiotic prior to hospitalization (Table 2).

**Table 2:** Anamnestic information.

Anamnesis	Origin of infection												
	Total		AP		PEI		PERI		MF		Other		p-value
	*n*	%	*n*	%	*n*	%	*n*	%	*n*	%	*n*	%	
Patients	384	100.0	180	100.0	99	100.0	23	100.0	33	100.0	49	100.0	
Duration of symptoms, mean ± s.d.	6.5	7.1	6	5.9	6.7	9.1	7.9	7	7.5	5.3	6.7	7	0.818
Refered from dentist	72	18.8	30	16.7	25	25.3	4	17.4	5	15.2	8	16.3	0.441
Antibiotic before hospitalization	251	65.4	115	63.9	68	68.7	13	56.5	27	81.8	28	57.1	0.172
Penicillin before hospitalization	136	35.4	67	37.2	39	39.4	8	34.8	13	39.4	9	18.4	0.112
Metronidazole before hospitalization	106	27.6	50	27.8	35	35.4	2	8.7	6	18.2	13	26.5	0.085
Penicillin + Metronidazole before hospitalization	51	13.3	28	15.6	18	18.2	0	0.0	6	18.2	3	6.1	0.134
Former incision and drainage	11	2.9	5	2.8	1	1.0	0	0.0	3	9.1	2	4.1	0.163
Initially misdiagnosis	58	15.1	27	15.0	11	11.1	10	43.5	3	9.1	7	14.3	0.002*

Test results of chi-square, Shapiro-Wilk, or Kruskal-Wallis with significant levels < 0.05 indicated by *.

AP: apical periodontitis; PEI: post-extraction infection; PERI: pericoronitis; MF: multiple foci; s.d.: standard deviation.

The origin of infection analyzed by type of tooth showed that molars, specifically mandibular molars, were the most frequent origin of infection in the five diagnosis groups (Table 3). Mandibular molars were related to 61.7% of the diseases from AP, 77.8% post-extraction infections, and 100% PERI cases.

**Table 3:** Dental origin of infection.

Type of tooth	Origin of infection									
	Total		AP		PEI		PERI		MF	
	*n*	%	*n*	%	*n*	%	*n*	%	*n*	%
Maxillary Incisor/Canine	8	2.1	8	4.4	0	0.0	0	0.0	0	0.0
Mandibular Incisor/Canine	4	1.0	3	1.7	0	0.0	0	0.0	1	1.2
Maxillary Premolar	6	1.6	6	3.3	0	0.0	0	0.0	0	0.0
Mandibular Premolar	16	4.2	12	6.7	3	3.0	0	0.0	1	1.2
Maxillary Molar	18	4.7	10	5.6	8	8.1	0	0.0	0	0.0
Mandibular Molar	218	56.8	111	61.7	77	77.8	23	100.0	7	8.5
Missing	114	29.7	30	16.7	11	11.1	0	0.0	73	89.0
Total	384	100.0	180	100.0	99	100.0	23	100.0	82	100.0

AP: apical periodontitis; PEI: post-extraction infection; PERI: pericoronitis; MF: multiple foci.

CT scans were available for 161 (41.3%) patients. As shown in Table 4, the localization of the abscess (mandible and/or maxilla) was significantly different between the diagnosis groups (*p* < 0.001). The analysis showed that if the abscess was in the maxilla, it was most likely due to AP. In contrast, there was a significant tendency for PEI to be more frequently found in the mandible according to the adjusted standardized residuals. If the infection was located in both the maxilla and the mandible, the cause was most likely to be MF.

**Table 4:** Localization of infection.

Anatomical location	Origin of infection											
	Total		AP		PEI		PERI		MF		Other	
	*n*	%	*n*	%	*n*	%	*n*	%	*n*	%	*n*	%
Mandibular	307	79.9	138	76.7	88	88.9	23	100.0	26	78.8	58	70.7
Maxillary	47	12.2	33	18.3	4	4.0	0	0.0	2	6.1	10	12.2
Mandibular + Maxillary	14	3.6	4	2.2	5	5.1	0	0.0	5	15.2	5	6.1
Missing	16	4.2	5	2.8	2	2.0	0	0.0	0	0.0	9	11.0
Total	384	100.0	180	100.0	99	100.0	23	100.0	33	100.0	82	100.0

AP: apical periodontitis; PEI: post-extraction infection; PERI: pericoronitis; MF: multiple foci.

A PR was available for 358 patient records (91.8%) (Table 5). More than 30% of the patients had between 2 and 10 apical lesions in the diagnosis groups AP and MF. There was a significant difference in the mean number of AP lesions (*p* < 0.001), where AP patients were overrepresented in the categories of 1 and 2–10, respectively, compared to the diagnosis groups (Table 5). In the analysis of marginal bone loss, the MF group had significantly more patients with severe periodontitis with <66% remaining marginal bone (*p* = 0.024). A significant difference was found in DMFT (*p* < 0.001) for AP patients, who were more likely to have 11–28 in DMFT according to the adjusted standardized residuals.

**Table 5:** Dental status.

		Origin of infection												
		Total		AP		PEI		PERI		MF		Other		p-value
		*n*	%	*n*	%	*n*	%	*n*	%	*n*	%	*n*	%	
Patients		384	100.0	180	100.0	99	100.0	23	100.0	33	100.0	49	100.0	
DMFT	0	28	7.3	21	11.7	1	1.0	4	17.4	0	0.0	2	4.1	<0.001*
	1–3	43	11.2	9	5.0	21	21.2	6	26.1	3	9.1	4	8.2	
	4–10	99	25.8	48	26.7	24	24.2	2	8.7	14	42.4	11	22.4	
	11–28	146	38.0	73	40.6	34	34.3	7	30.4	8	24.2	24	49.0	
Number of apical periodontitis	0	129	33.6	34	18.9	58	58.6	16	69.6	4	12.1	17	34.7	<0.001*
	1	79	20.6	48	26.7	13	13.1	2	8.7	6	18.2	10	20.4	
	2–10	108	28.1	69	38.3	9	9.1	1	4.3	15	45.5	14	28.6	
Mean remaining bone	≥80%	209	54.4	98	54.4	61	61.6	16	69.6	12	36.4	22	44.9	0.024*
	79% – 66%	44	11.5	24	13.3	4	4.0	1	4.3	7	21.2	8	16.3	
	<66%	66	17.2	31	17.2	15	15.2	2	8.7	6	18.2	12	24.5	

Test results of Chi-square with significant levels < 0.05 indicated by *.

AP: apical periodontitis; PEI: post extraction infection; PERI: Pericoronitis; MF: Multiple foci; DMFT: Decayed, Missing, Filled, teeth.

In the unadjusted analysis of the course of treatment during hospitalization, AP patients were more likely to have been handled using local anesthesia than patients with PEI (*p* = 0.005). Furthermore, there were significant differences in the need for tooth extractions during hospitalization (*p* < 0.001); patients with AP, PERI, and MF were more likely to have an extraction performed, and patients with PEI were less likely to, according to adjusted standardized residuals. The use of intravenous antibiotics significantly differed between the groups (*p* < 0.001). Fewer patients in the PERI and Other groups received treatment with intravenous antibiotics. The overall mean LOS at the hospital was 3.9 nights (Table 6). The mean LOS for the five groups was AP: 3.1 nights, PEI: 4 nights, PERI: 4.5, MF: 6.1, and Other: 4.7 nights. Outliers were not excluded from the analysis of LOS. The outliers were predominantly patients who developed CNF and were transferred to the intensive care unit. The analysis showed that 12 patients (3.1%) developed CNF, and there was a significant difference between the groups in this respect (*p* = 0.006), with the MF group more frequently developing CNF.

**Table 6:** Course of hospitalization.

Characteristics	Origin of infection												*p-value*
	Total		AP		PEI		PERI		MF		Other		
	*n*	%	*n*	%	*n*	%	*n*	%	*n*	%	*n*	%
Patients	384	100.0	180	100.0	99	100.0	23	100.0	33	100.0	49	100.0	
1st day CRP, mean ± s.d.	133.1	105.2	124.8	100.3	138.4	109.8	114.0	107.3	181.6	106.1	126.8	107.3	0.089
1st day LEU, mean ± s.d.	13.6	5.1	13.3	4.7	13.5	5.2	11.7	4.9	16.2	6.2	13.4	5.5	0.032*
Local anesthesia	57	14.8	38	21.1	4	4.0	4	17.4	4	12.1	7	14.3	0.005*
General anesthesia	269	70.1	117	117.0	70	70.7	18	78.3	24	72.7	40	81.6	0.206
Extraction	185	48.2	109	60.6	14	14.1	18	78.3	21	63.6	23	46.9	<0.001*
Insicion and drainage	268	69.8	117	65.0	71	71.7	17	73.9	23	69.7	35	71.4	0.099
One-time surgery	241	62.8	109	60.6	60	60.6	17	73.9	18	54.5	37	75.5	0.052
Two-time surgery	24	6.3	7	3.9	8	8.1	1	4.3	6	18.2	2	4.1	
Three-time surgery	5	1.3	1	0.6	3	3.0	0	0.0	0	0.0	1	2.0	
IV- antibiotics	287	74.7	143	79.4	80	80.8	15	65.2	25	75.8	24	49.0	<0.001*
Shift in IV antibiotics	48	12.5	15	8.3	12	12.1	3	13.0	7	21.2	11	22.4	0.042*
LOS, mean ± s.d.	3.9	4.5	3.1	2.2	4.2	4.4	4.5	7.1	6.1	6.6	4.7	0.9	0.002*
Development of CNF	12	3.1	2	1.1	2	2.0	2	8.7	4	12.1	2	4.1	0.006*
AB: Antibiotic resistance	165	43.0	69	38.3	46	46.5	9	39.1	20	60.6	21	42.9	0.724
Dysesthesia	23	6.0	7	3.9	8	8.1	1	4.3	4	12.1	3	6.1	0.403

Test results of chi-square, Shapiro-Wilk, or Kruskal-Wallis with significant levels < 0.05 indicated by *.

LEU: Leucocytes; IV: Intravenous; CNF: Cervical necrotizing fasciitis; LOS: Length of stay; CRP: C-reactive protein; AP: apical periodontitis; PEI: post-extraction infection; PERI: pericoronitis; MF: multiple foci; s.d.: standard deviation;

* : Significant.

There was no significant difference C-reactive protein (CRP) on the first day of hospitalization between the five diagnosis groups (*p* = 0.089) (Table 6). Leukocyte count was found to be significantly higher in the MF group compared to four other groups (*p* = 0.032).

### Logistic regression

In total, 260 patients were hospitalized for <4 days and 124 for ≥4 days. Older age, hypertension, alcohol consumption, penicillin allergy, and origin of infection were all significantly associated with ≥4 days (*p* < 0.05). These variables were included in the binary logistic regression model (Table 7). According to the Omnibus Tests of Model Coefficients and a Hosmer and Lemeshow test, the model’s goodness of fit predicted 11% of the variability in the data.

**Table 7:** Logistic regression model predicting likelihood of LOS ≥4 days.

Variables	*B*	SE	Wald	*df*	*p*	Odds ratio	95% CI for Odds ratio	
							Lower	Upper
Age	0.01	0.01	0.72	1.00	0.40	1.01	0.99	1.02
Alcohol consumption	0.83	0.31	7.21	1.00	0.01	2.30	1.25	4.21*
Penicillin allergy	-0.08	0.51	0.03	1.00	0.87	0.92	0.34	2.49
Hypertension	1.06	0.43	5.98	1.00	0.01	2.89	1.23	6.76*
AP	0.00	0.00	9.54	4.00	0.05	ref.	ref.	ref.*
PEI	0.32	0.33	0.95	1.00	0.33	1.38	0.72	2.65
PERI	-0.35	0.71	0.24	1.00	0.62	0.70	0.18	2.84
MF	1.41	0.48	8.62	1.00	<0.01	4.12	1.60	10.58*
Other	0.36	0.42	0.75	1.00	0.39	1.44	0.63	3.28
Constant	-2.01	0.43	21.98	1.00	0.00	0.13		

LOS: length of stay; B: regression coefficient (B); SE: standard error; Wald: Wald statistic; df: degrees of freedom; *p*: *p*-value; CI: confidence interval; AP: apical periodontitis; PEI: post-extraction infection; PERI: pericoronitis; MF: multiple foci;

* : Significant.

In the model, alcohol consumption (*p* = 0.01), hypertension (*p* = 0.01), and having MF (*p* ≤ 0.01) were found to have a statistically significant effect on the risk of LOS ≥4 days. Patients who reported the use of alcohol had a 2.30 (CI [confidence interval] = 1.25; 4.21) odds ratio for LOS ≥4 days, and patients who suffered from hypertension had a 2.89 (CI = 1.23; 6.76) odds ratio for LOS ≥4 days. Patients with MF had a 4.12 (CI = 1.60; 10.58) odds ratio for LOS ≥4 days.

### Multiple correspondence analysis

**Figure 2:**
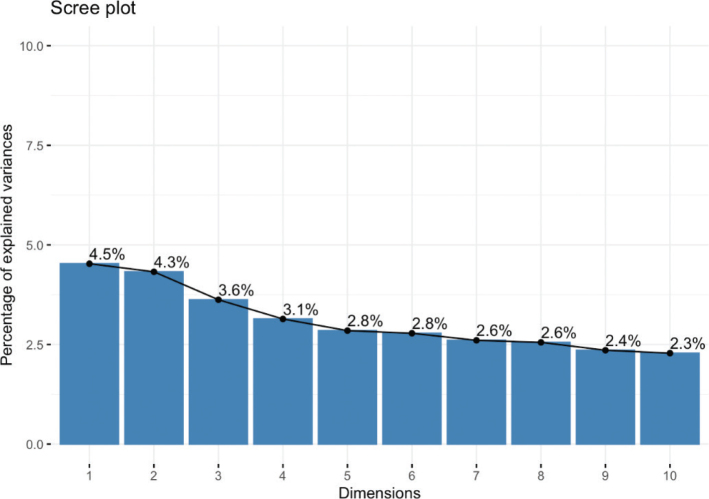
Scree plot. This figure illustrates the 10 variables (1–10) which contribute to the highest degree of explanation of the variance in the data.

**Figure 3:**
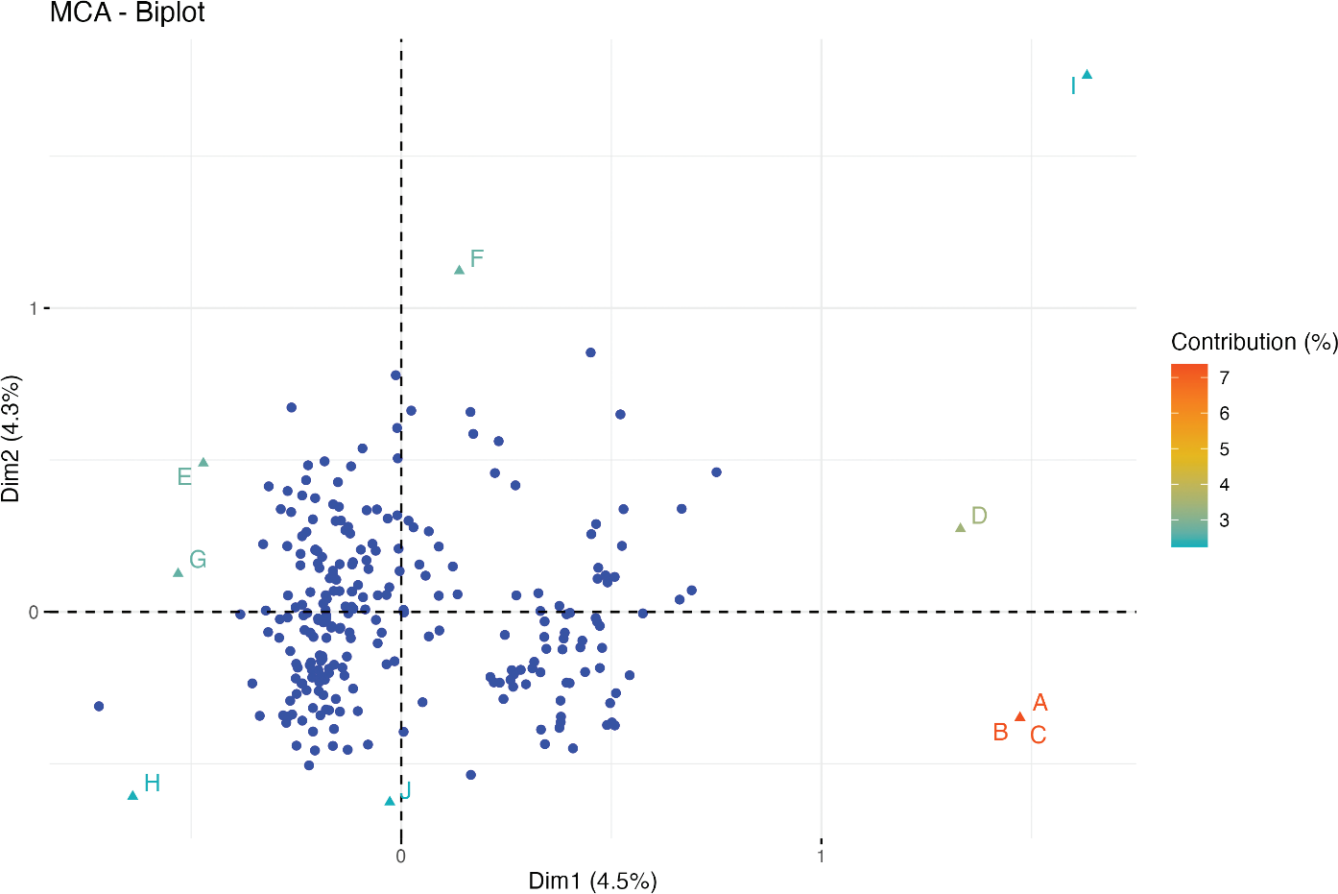
MCA – first biplot. This figure illustrates the distribution of patients (blue marks) in the MCA, based on the two most explanatory dimensions. The letters (A to I) represent the nine variables that have the greatest degree of contribution to distribution in the MCA. The color of the variables represents the degree of contribution. MCA: multiple correspondence analysis; Dim1: Dimension 1; Dim2: Dimension 2; A: No operation in general anesthesia; B: Not possible with general anesthesia; C: no drain in general anesthesia; D: Operation in local anesthesia; E: Tooth extraction in local anesthesia; F: No treatment with cefuroxime and metronidazole; G: Operation in general anesthesia; H: No tooth extraction in general anesthesia; I: No treatment with intravenous antibiotics.

**Figure 4:**
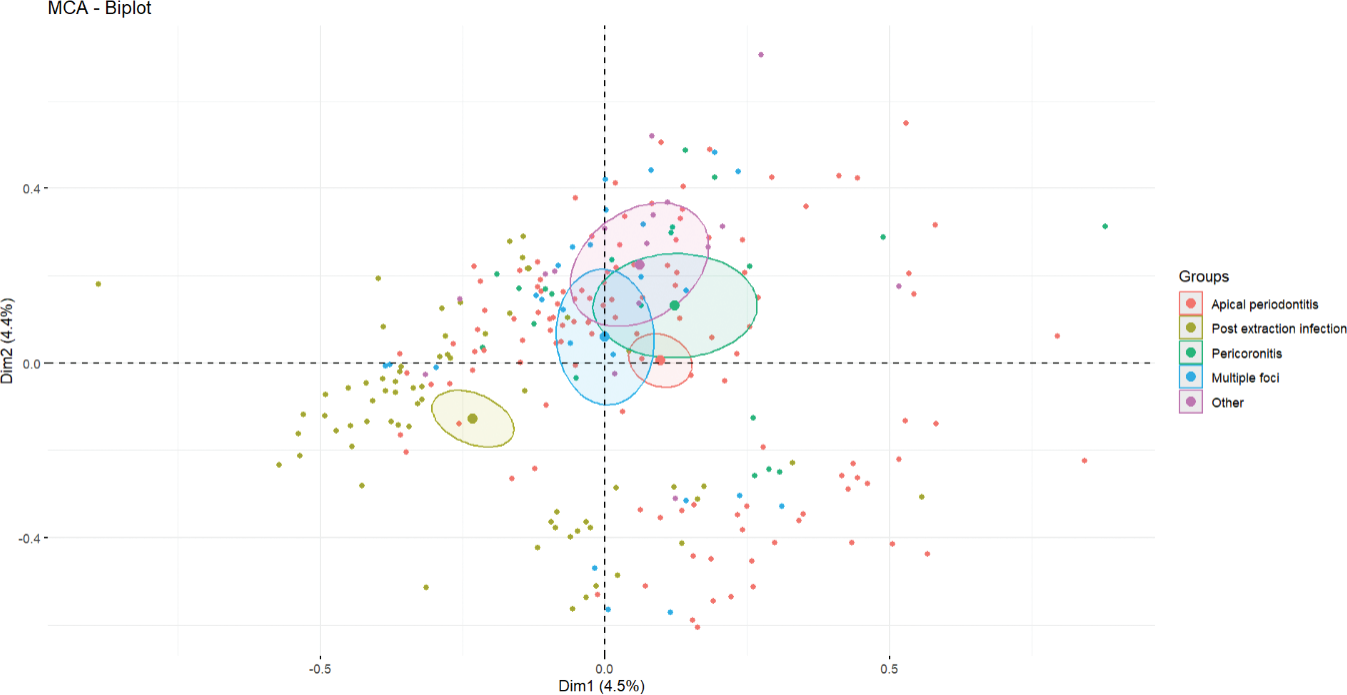
MCA – second biplot. This figure illustrates the distribution of patients in the MCA, divided into origin of infection (red, dark green, light green, blue, purple). The colored shades show the distribution based on the origin of the infection. MCA: multiple correspondence analysis; Dim1: Dimension 1; Dim2: Dimension 2.

After missing data were excluded, 249 patients and 60 variables were left for MCA. The proportion of variability explained by each of the new dimensions is shown in the Scree plot (Figure 2). The first dimension explained 4.5% of the variability in the data, and the first 10 dimensions cumulatively explained 31% of the variability in the data. Patients were clustered in two distinct groups along the first dimension (Figure 3). However, these clusters did not overlap with the origin of infection (Figure 4).

Rather, the two clusters were primarily determined by variables describing treatment at the hospital. I: Patients undergoing surgery in general anesthesia, some of whom had tooth extraction performed in general anesthesia, and some of whom had not. Some did not receive standard treatment with cefuroxime and metronidazole during hospitalization. II: The second group did not undergo surgery in general anesthesia, but some patients in this group underwent surgery in local anesthesia. Treatment before hospitalization seems to be of importance in the distribution of the patients.

## Discussion

This study showed that patients with different origins of infection are sparsely different regarding variables and medical history and only to a low degree during the course of their hospital stay. Factors that were found to increase the risk of prolonged LOS were alcohol consumption, hypertension, and MF. Furthermore, the results showed that patients suffering from SOI rarely receive adequate dental and/or medical treatment before hospitalization, underlined by the low number of patients who have received antibiotics and/or incision and drainage.

The distribution of odontogenic origin leading to SOI in this study is comparable to findings from other studies [18]. The categorization of patients based on the origin of infection showed little to no ability to detect differences in variables. In terms of background information, significant differences between diagnosis groups were found for age, number of comorbidities, and dental status. In general, the patients had poor dental status regarding dental decay and the number of periapical lesions. Unlike previous studies, most patients in the present study were females [8, 9, 11, 13, 16, 19–30]. The origin of infection showed no significant difference in sex.

In the present cohort of patients, the proportion of patients with immunodeficiency diseases and with comorbidities in general was low, in accordance with findings from other studies on SOI [8, 11, 13, 16, 17, 23, 26]. This challenges the classic perception of a patient with SOI as an immunocompromised patient predisposed to infection. Patients in the AP, MF, and Other groups had significantly more comorbidities than patients with PEI and PERI. The patients in these three groups were significantly older than patients with PEI and PERI and may thus have been more prone to accumulate comorbidities over time.

Initial findings in differences in LOS between the diagnosis groups were, in the logistic model, found to be confounded by hypertension and alcohol consumption. The mean LOS of 3.9 days is relatively low compared to other SOI studies, which vary between 3.3 and 19.9 days [8, 9, 13, 15, 22–25, 31]. Sex showed no significant effect on LOS, which corresponds with previous findings [8]. Other studies have shown that concurrent DM and increasing age are risk factors for prolonged hospital stays and the development of deep neck infections during treatment for oral and maxillofacial infections [23, 32]. The mean age observed in this study, 43.9 years, which is similar to that of other studies, which have mean ages ranging between 37 and 50 years [8, 9, 11, 15, 16, 19–22, 25, 26, 28, 31, 33]. As was also observed in this study, age has previously been found to affect the LOS. In multivariate models made in previous studies, the effect of age is significant but minimal, with 0.007 to 0.06 days per additional year [8, 26, 34, 35]. In general, health behaviors such as smoking did not affect LOS, which have also been found in similar studies [9, 13, 16, 26]. Patients reporting regular alcohol consumption, on the other hand, had a significantly longer LOS.

Patients with SOI often report having had severe symptoms persisting for several days before hospitalization [21]. In theory, the delay in definitive care can worsen the condition [21, 24]. The number of days with symptoms aligned with previous studies [21]. We found that patients with MF had a LOS significantly higher than the other diagnosis groups. Patients experienced a relatively high number of days with symptoms indicating that barriers might exist for the patient to receive appropriate dental treatment. This hypothesis could be supported by the fact that only 1/3 of patients were referred by a dentist, with the majority of study participants being referred to treatment from emergency departments. In this study, relatively few patients had received incision and drainage before hospitalization, which aligns with previous findings [21].

At the time this study was conducted, the official Danish guidelines for antibiotic therapy of patients suffering from SOI were a combination therapy, including penicillin in combination with metronidazole. The results of this study showed that very few patients received antibiotic treatment following the Danish guidelines. A substantial number of patients received no antibiotic treatment at all before hospitalization. At the hospital clinic, the standard antibiotic therapy was IV cefuroxime in combination with IV metronidazole. The use of cefuroxime as a primary choice of antibiotics, as in this current hospital setting, can be questioned as critical. Cefuroxime is, according to Danish guidelines, perceived as a critically important antibiotic; future studies should focus on the necessity of using cefuroxime as a first choice.

This study found that patients suffering from SOI, in general, had poor dental status, did not receive incision and drainage of the abscess, and did not receive adequate antibiotic treatment before hospitalization. All these factors indicate inadequate pre-hospital care. Studies have shown that hospitalization with SOI is associated with a relatively high economic burden [22, 26]. Regardless of the different approaches to financing healthcare between countries, there is a common interest in minimizing the number of hospitalizations across the globe, as this is beneficial both from a socioeconomic and patient point of view. From a public health perspective, it would be desirable to place dentistry in a more central position in the healthcare system in regard to the treatment of odontogenic infections and consequently in the prevention of SOI. Future studies should collect and analyze anamnestic information for AP and PEI groups to detect differences in treatment modalities. Furthermore, future studies should focus on patients suffering from dental neglect, which is a risk factor for developing SOI, and investigate structural barriers preventing patients with SOI from receiving sufficient pre-hospital care.

This study was limited by its design, being a cross-sectional study based on retrospective data from medical record. The study design resulted in missing values in some variables, especially regarding anamnestic information. This may increase the risk of skewed data affecting the results of the MCA and the regression models. This was underlined by the MCA’s first 10 dimensions, only cumulatively capturing 31% of the variability in the data. A prospective study design would ensure a more systematic data collection, which could facilitate the generation of more robust models.

## Conclusion

The four most prevalent infectious origins underlying SOI are AP, PEI, MF, and PERI. No clusters of patients that align with the origin of infection could be identified using a dimensionality reduction technique. Patients suffering from SOI had, in general, not received sufficient dental treatment. LOS at the hospital did differ between the origin of infection and patients with MF had a longer LOS. There may be a substantial preventive effect if treatment of the causative condition, incision and drainage, and supportive antibiotic treatment are initiated in general dental practice.

## References

[1] Aziz Z, Aboulouidad S, Bouihi ME, et al. Odontogenic cervico-facial cellulitis during pregnancy: about 3 cases. Pan Afr Med J. 2020;36:258. 10.11604/pamj.2020.36.258.24864. 33014254 PMC7519797

[2] Jespersen FVB, Hansen SU, Jensen SS, et al. Cerebral abscesses with odontogenic origIn: a population-based cohort study. Clin Oral Investig. 2023;27(7):3639–3648. 10.1007/s00784-023-04976-6. PMC10329578 37002439

[3] Hansen SU, Jespersen FVB, Markvart M, et al. Characterization of patients with odontogenic necrotizing soft tissue infections in the head and neck area. A retrospective analysis. Acta Odontol Scand. 2024;82(1):40–47. 10.1080/00016357.2023.2254389. 37688516

[4] Guzmán-Letelier M, Crisosto-Jara C, Diaz-Ricouz C, et al. Severe odontogenic infection: an emergency. Case report. J Clin Exp Dent. 2017;9(2):e319–e324. 10.4317/jced.53308. 28210456 PMC5303338

[5] Rega AJ, Aziz SR, Ziccardi VB. Microbiology and antibiotic sensitivities of head and neck space infections of odontogenic origin. J Oral Maxillofac Surg. 2006;64(9):1377–1380. 10.1016/j.joms.2006.05.023. 16916672

[6] De Moor RJ, Hommez GM, De Boever JG, et al. Periapical health related to the quality of root canal treatment in a Belgian population. Int Endod J. 2000;33(2):113–120. 10.1046/j.1365-2591.2000.00295.x. 11307451

[7] Meirinhos J, Martins JNR, Pereira B, et al. Prevalence of apical periodontitis and its association with previous root canal treatment, root canal filling length and type of coronal restoration – a cross-sectional study. Int Endod J. 2020;53(4):573–584. 10.1111/iej.13256. 31749154

[8] Christensen BJ, Racha D, Hinkle R, et al. Risk factors for reoperation in patients hospitalized for odontogenic infections. J Oral Maxillofac Surg. 2021;79(1):141–151. 10.1016/j.joms.2020.06.032. 32717213

[9] Pham Dang N, Delbet-Dupas C, Mulliez A, et al. Five predictors affecting the prognosis of patients with severe odontogenic infections. Int J Environ Res Public Health. 2020;17(23):8917. 10.3390/ijerph17238917. 33266250 PMC7730806

[10] Seppänen L, Rautemaa R, Lindqvist C, et al. Changing clinical features of odontogenic maxillofacial infections. Clin Oral Investig. 2010;14(4):459–465. 10.1007/s00784-009-0281-5. 19449042

[11] Sánchez R, Mirada E, Arias J, et al. Severe odontogenic infections: epidemiological, microbiological and therapeutic factors. Med Oral Patol Oral Cir Bucal. 2011;16(5):e670–e676. 10.4317/medoral.16995. 20711116

[12] Kim MK, Nalliah RP, Lee MK, et al. Factors associated with length of stay and hospital charges for patients hospitalized with mouth cellulitis. Oral Surg Oral Med Oral Pathol Oral Radiol. 2012;113(1):21–28. 10.1016/j.tripleo.2011.01.012. 22677688

[13] Fu B, McGowan K, Sun JH, et al. Increasing frequency and severity of odontogenic infection requiring hospital admission and surgical management. Br J Oral Maxillofac Surg. 2020;58(4):409–415. 10.1016/j.bjoms.2020.01.011. 31987682

[14] Velhonoja J, Lääveri M, Soukka T, et al. Deep neck space infections: an upward trend and changing characteristics. Eur Arch Otorhinolaryngol. 2020;277(3):863–872. 10.1007/s00405-019-05742-9. 31797041 PMC7031181

[15] Seppänen L, Lauhio A, Lindqvist C, et al. Analysis of systemic and local odontogenic infection complications requiring hospital care. J Infect. 2008;57(2):116–122. 10.1016/j.jinf.2008.06.002. 18649947

[16] Furuholm J, Rautaporras N, Uittamo J, et al. Health status in patients hospitalised for severe odontogenic infections. Acta Odontol Scand. 2021;79(6):436–442. 10.1080/00016357.2021.1876916. 33502919

[17] Rydén L, Buhlin K, Ekstrand E, et al. Periodontitis increases the risk of a first myocardial infarction: a report from the PAROKRANK study. Circulation. 2016;133(6):576–583. 10.1161/CIRCULATIONAHA.115.020324. 26762521

[18] Furuholm J, Uittamo J, Rautaporras N, et al. Are there differences between dental diseases leading to severe odontogenic infections requiring hospitalization? A retrospective study. Quintessence Int. 2022;53(6):484–491. 35274510 10.3290/j.qi.b2793183

[19] Hwang T, Antoun JS, Lee KH. Features of odontogenic infections in hospitalised and non-hospitalised settings. Emerg Med J. 2011;28(9):766–769. 10.1136/emj.2010.095562. 21045219

[20] Bowe CM, O’Neill MA, O’Connell JE, et al. The surgical management of severe dentofacial infections (DFI) – a prospective study. Ir J Med Sci. 2019;188(1):327–331. 10.1007/s11845-018-1802-5. 29700733

[21] Katoumas K, Anterriotis D, Fyrgiola M, et al. Epidemiological analysis of management of severe odontogenic infections before referral to the emergency department. J Craniomaxillofac Surg. 2019;47(8):1292–1299. 10.1016/j.jcms.2019.05.002. 31331847

[22] Heim N, Warwas FB, Wiedemeyer V, et al. The role of immediate versus secondary removal of the odontogenic focus in treatment of deep head and neck space infections. A retrospective analysis of 248 patients. Clin Oral Investig. 2019;23(7):2921–2927. 10.1007/s00784-018-02796-7. 30623306

[23] Huang TT, Tseng FY, Liu TC, et al. Deep neck infection in diabetic patients: comparison of clinical picture and outcomes with nondiabetic patients. Otolaryngol Head Neck Surg. 2005;132(6):943–947. 10.1016/j.otohns.2005.01.035. 15944569

[24] Jundt JS, Gutta R. Characteristics and cost impact of severe odontogenic infections. Oral Surg Oral Med Oral Pathol Oral Radiol. 2012;114(5):558–566. 10.1016/j.oooo.2011.10.044. 22819453

[25] Zirk M, Buller J, Goeddertz P, et al. Empiric systemic antibiotics for hospitalized patients with severe odontogenic infections. J Craniomaxillofac Surg. 2016;44(8):1081–1088. 10.1016/j.jcms.2016.05.019. 27369813

[26] Gams K, Shewale J, Demian N, et al. Characteristics, length of stay, and hospital bills associated with severe odontogenic infections in Houston, TX. J Am Dent Assoc. 2017;148(4):221–229. 10.1016/j.adaj.2016.11.033. 28129825

[27] Rastenienė R, Pūrienė A, Aleksejūnienė J, et al. Odontogenic maxillofacial infections: a ten-year retrospective analysis. Surg Infect (Larchmt). 2015;16(3):305–312. 10.1089/sur.2013.264. 26046244

[28] Uittamo J, Löfgren M, Hirvikangas R, et al. Severe odontogenic infections: focus on more effective early treatment. Br J Oral Maxillofac Surg. 2020;58(6):675–680. 10.1016/j.bjoms.2020.04.004. 32507644

[29] Bertossi D, Barone A, Iurlaro A, et al. Odontogenic orofacial infections. J Craniofac Surg. 2017;28(1):197–202. 10.1097/SCS.0000000000003250. 27930461

[30] Stathopoulos P, Igoumenakis D, Shuttleworth J, et al. Predictive factors of hospital stay in patients with odontogenic maxillofacial infections: the role of C-reactive protein. Br J Oral Maxillofac Surg. 2017;55(4):367–370. 10.1016/j.bjoms.2016.11.004. 27876162

[31] Opitz D, Camerer C, Camerer DM, et al. Incidence and management of severe odontogenic infections-a retrospective analysis from 2004 to 2011. J Craniomaxillofac Surg. 2015;43(2):285–289. 10.1016/j.jcms.2014.12.002. 25555896

[32] Park J, Lee JY, Hwang DS, et al. A retrospective analysis of risk factors of oromaxillofacial infection in patients presenting to a hospital emergency ward. Maxillofac Plast Reconstr Surg. 2019;41(1):49. 10.1186/s40902-019-0238-9. 31815113 PMC6872703

[33] Sato FR, Hajala FA, Freire Filho FW, et al. Eight-year retrospective study of odontogenic origin infections in a postgraduation program on oral and maxillofacial surgery. J Oral Maxillofac Surg. 2009;67(5):1092–1097. 10.1016/j.joms.2008.09.008. 19375023

[34] Boffano P, Roccia F, Pittoni D, et al. Management of 112 hospitalized patients with spreading odontogenic infections: correlation with DMFT and oral health impact profile 14 indexes. Oral Surg Oral Med Oral Pathol Oral Radiol. 2012;113(2):207–213. 10.1016/j.tripleo.2011.02.006. 22677738

[35] Heim N, Jürgensen B, Kramer FJ, et al. Mapping the microbiological diversity of odontogenic abscess: are we using the right drugs? Clin Oral Investig. 2021;25(1):187–193. 10.1007/s00784-020-03350-0. 32472254

